# Reduced pollinator service in small populations of *Arabidopsis lyrata* at its southern range limit

**DOI:** 10.1007/s00442-022-05237-1

**Published:** 2022-09-02

**Authors:** Darío Sánchez-Castro, Georg Armbruster, Yvonne Willi

**Affiliations:** Department of Environmental Sciences, University of Basel, 4056 Basel, Switzerland

**Keywords:** Allee effect, Density, Latitudinal gradient, Pollination, Preference

## Abstract

Even though a high fraction of angiosperm plants depends on animal pollinators for sexual reproduction, little is known how pollinator service changes across the ranges of plant species and whether it may contribute to range limits. Here, we tested for variation in pollinator service in the North American *Arabidopsis lyrata* from its southern to northern range edge and evaluated the driving mechanisms. We monitored insect pollinators using time-lapse cameras in 13 populations over two years and spotted 67 pollinating insect taxa, indicating the generalist nature of this plant-pollinator system. Pollinator service was highest at intermediate local flower densities and higher in large compared to small plant populations. Southern populations had generally smaller population sizes, and visitation rate and pollination ratio decreased with latitude. We also found that pollinator visitation was positively correlated with the richness of other flowering plants. This study indicates that plant populations at southern range edges receive only marginal pollinator service if they are small, and the effect of lower pollination is also detectable within populations across the range when the local flower density is low. Results, therefore, suggest the potential for an Allee effect in pollination that manifests itself across spatial scales.

**Supplementary Information:**

The online version contains supplementary material available at 10.1007/s00442-022-05237-1.

## Introduction

Species’ range limits, when not caused by dispersal limitation, should generally reflect the limits of the ecological niche. In many species, niches and ranges seem to be limited by climatic factors such as temperature and precipitation (Sexton et al. 2009). In line, species’ distribution modelling indicates that a handful of climatic variables can often explain distribution limits rather well (e.g. Normand et al. 2009; Lee-Yaw et al. 2016). However, biotic interactions have been considered less often in distribution modelling, and in the study of species’ distribution limits more generally (Sexton et al. 2009). This neglect is not justified because empirical studies show that biotic interactions affect species persistence. Examples include interspecific competition (Jankowski et al. 2010; Stanton-Geddes et al. 2012), host–parasite and host–pathogen interactions (Briers 2003; Coates et al. 2017), and herbivory (Galen 1990; Benning and Moeller 2019). Mutualistic interactions are also known to affect species persistence, especially the one between plants and their pollinators (Stone and Jenkins 2008; Chalcoff et al. 2012; Moeller et al. 2012). Here, we explored variation in pollinator service across the distribution of a plant species, and the mechanisms by which pollinators may contribute to range limits.

Pollinator service is especially important for plant persistence as 80% of all temperate-zone flowering plant species rely, at least to some extent, on animals for pollination (Ollerton et al. 2011). At range edges, reduced pollinator service might constrain the abundance of plants that need animals as pollen vectors for reproduction (Gaston 2009). Indeed, population persistence is commonly reduced at range edges. A meta-analysis of transplant experiments with sites across and beyond range limits, mostly on plants, revealed that lifetime performance declined beyond the range in 83% of studies (Hargreaves et al. 2014). The decline seems affected by a change in climatic conditions beyond the edge (Lee-Yaw et al. 2016), but biotic interactions such as a lack of suitable pollinators could also contribute to range limits. Variation in pollinator service across the distribution of plant species can be related to climatic conditions that favour the activity of pollinators (Chalcoff et al. 2012; Moeller et al. 2012), but pollinator service could also vary due to floral attractiveness and pollinator preferences. For example, as climatic conditions deteriorate toward range limits, possibly together with habitat availability or habitat quality, population size, local flower density, flower attractiveness or the richness of flowering plant species may decrease. Below we discuss in detail the mechanisms potentially reducing pollinator service and their relation with the distribution of a plant species.

One mechanism that may reduce pollinator service at a plant’s range edge involves low plant abundance. Observations suggested that the abundance of an organism declines toward the edges, presumably because habitat suitability decreases (Brown 1984). The so-called ‘abundant-centre hypothesis’ is broadly supported by a recent study documenting a decline in the density of individuals within populations and of populations from the centre to the edges of species’ distributions (Pironon et al. 2017). Lower local and regional densities of plants at range edges may lower their attractiveness to pollinators because pollinators commonly exhibit a preference for patches with a high density of flowering plants (reviewed by Ohashi and Yahara 1999; Stone and Jenkins 2008; Elliott and Irwin 2009). This hypothesis describes a potential Allee effect (Courchamp et al. 1999), namely that pollinator service is lower in plant populations of low density and small size that may affect seed set.

A second mechanism is reduced floral attractiveness at range edges. Animal-pollinated plants can sometimes enhance attractiveness to pollinators, e.g. by producing more flowers per plant, or larger flowers (e.g. Klinkhamer and De Jong 1990; Grindeland et al. 2005). However, investments in floral display may be costly and hard to achieve if the environment is marginal and provides limited resources. Furthermore, plants of range-edge populations may often have reduced individual performance because of mutation accumulation due to past range expansion or long-term isolation combined with enhanced genetic drift (Willi et al. 2018; Willi and Van Buskirk 2019; Perrier et al. 2020). Perrier et al. showed that a decline in performance of *Arabidopsis lyrata* of range edges was associated with reduced flower production. Moreover, floral attractiveness may be lower at range edges because of a transition in the mating system from outcrossing to selfing (Morgan and Wilson 2005; Moeller 2006). Higher rates of self-compatibility and selfing have been noted in range-edge populations (e.g. Griffin and Willi 2014), and such a shift in the mating system may be associated with evolutionary changes in floral morphology such as a reduction in flower size (Darling et al. 2008; Dart et al. 2012). Hence, reduced attractiveness of flowers due to ecological or genetic reasons may be another possibility for low pollinator service at range edges.

A third likely mechanism is related to the richness of flowering plant species and the diversity of resources offered to pollinators. Previous studies have reported a positive relationship between the diversity of flower types among co-occurring plants and the diversity and abundance of pollinators (e.g. Biesmeijer et al. 2006; Lázaro and Totland 2010). The richness and abundance of other flowering plants increase the pool of resources available to pollinators and therefore attract a broader diversity of insect visitors. If conditions at the edge of a species’ range become marginal for several plant species and the plant community is therefore less diverse, pollinators might avoid visiting them.

Finally, a fourth mechanism for reduced pollinator service at a plant’s range edge is that climatic conditions may be unsuitable for pollinator activity. As conditions are expected to become climatically harsher toward the edges, guilds of pollinators that are to some extent specialized on a community of plants may also decline in abundance. It is well known that pollinator abundance and metabolic activity are highly affected by temperature (Herrera 1990; Hillyer and Silman 2010; Rader et al. 2012; Knop et al. 2018). Therefore, an environmental gradient that limits plant populations may have similar consequences for the pollinator assembly (e.g. Battisti et al. 2006).

In this study, we tested whether pollinator service decreased toward the range edges of a plant’s distribution (research question I) and explored the mechanisms at play (research question II). Our study organism was the short-lived perennial *Arabidopsis lyrata* subsp*. lyrata* in North America, which has been the subject of ongoing research focussing on the ecological and evolutionary causes of distribution limits (Lee-Yaw et al. 2018; Willi et al. 2018, 2020; Perrier et al. 2020; Sánchez-Castro et al. 2022). We assessed daily visitation of flowers by pollinators in 13 populations across a latitudinal gradient of 1100 km in the eastern United States, including replicate populations at the southern limit, in the centre of the range, and at the northern range limit. We quantified and identified pollinators using time-lapse cameras in each population, and tested for support for the four potential mechanisms of pollinator decline by relating pollinator data to population and site characteristics.

## Material and methods

### Study organism

**Fig. 1:**
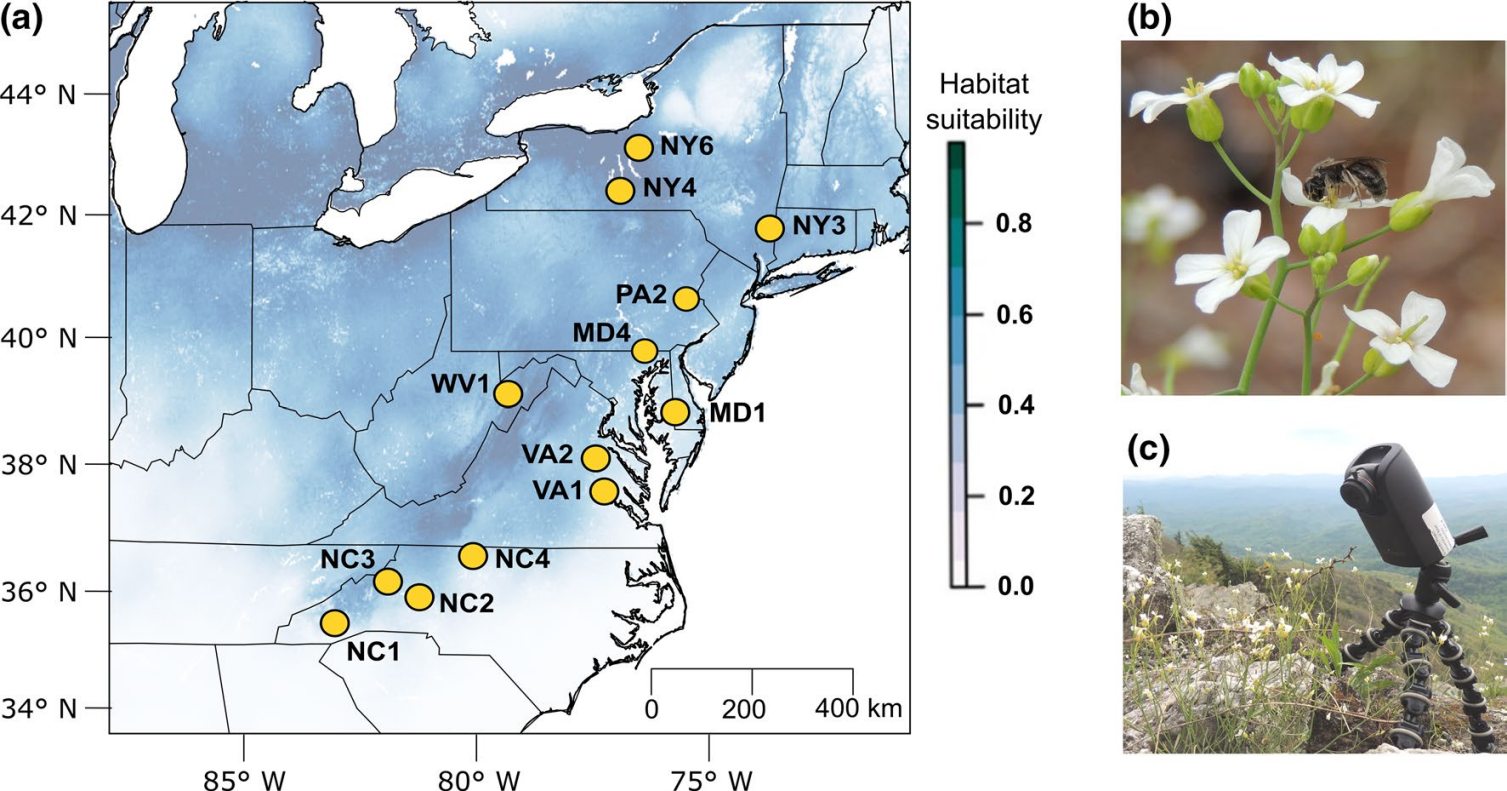
**a** Map of eastern North America with the 13 *Arabidopsis lyrata* populations studied for pollinator service, and **b** images of *A. lyrata* flowers with a wild bee visiting and **c** a time-lapse camera monitoring a patch of flowers in the field. In panel **a**, populations are indicated by dots and a three-digit abbreviation (Table S1, the two letters stand for the state in the US, and the number is the latitudinal position within the state). Shades of blue indicate habitat suitability for the species based on niche modelling, where minimum temperature in early spring and precipitation of the wettest quarter were the climatic variables that predicted the presence of the species best

*Arabidopsis lyrata* comprises two subspecies that together have a circumpolar distribution: *A. lyrata* subsp. *petraea* of mostly northern Eurasia, and *A. lyrata* subsp. *lyrata* of central and eastern North America (Schmickl et al. 2010). The North American subspecies (hereafter abbreviated *A. lyrata*) has a well-defined distribution in the US and Canada. One ancestral genetic cluster occurs from North Carolina to the state of New York in the east, the other from Missouri to south-western Ontario in the Midwest (Willi and Määttänen 2010; Willi et al. 2018). Populations typically occur on sand dunes, rocky outcrops, or on sandy or rocky riverbanks and shorelines. In the Appalachians, plants grow on poor soils of coniferous leaf litter, under evergreen trees dominated by Virginia pine (*Pinus virginiana)* and Eastern Red Cedar (*Juniperus virginiana)*, or they grow on moss on top of bedrock. For this study, a total of 13 populations were monitored on a latitudinal gradient of 1100 km along the Appalachians, from North Carolina to upstate New York (Fig. 1a, Table S1).

*A. lyrata* is mostly outcrossing and insect-pollinated. However, some populations at range edges are self-compatible and predominantly selfing (Griffin and Willi 2014), though most of them are not autonomously selfing but require pollinators for self-pollen deposition. Plants produce basal rosettes with inflorescences emerging from about mid-April to mid-June in eastern populations (Fig. 1b). Both the number of inflorescences and flowers per inflorescence vary considerably among populations and with the age of the plant. Flowers have white petals and nectar discs at the base of the stamens. In *A. lyrata* subsp. *petraea*, volatiles were shown to be emitted from the petals during daytime, with a peak around midday (Abel et al. 2009). A previous study on one population on Isle Royale, Michigan, found that the dominant pollinators were syrphid flies (Edwards et al. 2019).

### Pollination records

The study of pollinators in the field has been typically centred on personal observations (e.g. Peckham and Peckham 1905; Rafferty and Ives 2011; Hargreaves et al. 2015). Here we used time-lapse cameras (TLC 200 Pro HDR, Brinno, Taipei City, Taiwan; Fig. 1c; Edwards et al. 2015) taking pictures of flowers at short intervals as an alternative to record pollinators. This approach offers several advantages: simultaneous spatial and temporal sampling can be increased without intensifying manpower; the effect of humans on insect behaviour is minimized; and the identification of pollinators based on images and behavioural movements may decrease detection and identification problems. The cameras provide enough precision to identify and quantify flower visitors independently of the flower morphology or insect group (Edwards et al. 2015).

In each population of *A. lyrata*, 10–12 cameras recorded separate flower patches for three days during 12 h, from 8 am to 8 pm, at an interval of 3 s (see Table S1 for detailed observation period and patches recorded). The 3-s interval was shown to detect 90% of all visits (Edwards et al., 2015). As the abundance of insect visitors is highly affected by temperature, wind and precipitation (Cruden 1972; Roubik 1989), observations were carried out only when the weather was sunny and the sky was mostly clear. Monitoring was performed during the period of full bloom, from mid-April in the south to early June in the north, for two consecutive years (2018 and 2019). Two populations were monitored in both years.

Videos were examined with the *Quick Time Player* programme (Apple, Cupertino, CA, USA). Visits were considered only if there was direct contact of the insect with the pistil or stamens of the flower. We identified insects to the lowest taxonomic unit given the quality of the images, using Kits et al. (2008), Miranda et al. (2013), and Skevington et al. (2019) as identification keys. If the image was blurry and the pollinator unrecognizable, the taxon was categorized as “unidentifiable”. Therefore, not all visits were identified at the same taxonomic depth. Some groups—especially in the Hymenoptera—were split into categories based on characters such as morphology, size, and colour pattern. We discarded from the analysis members of the Formicidae (ants) because their contribution to pollination is minimal (Junker et al. 2007). Curculionidae (weevils) were observed in one of the patches of a southern population, but not considered because of their small size and difficulties spotting them. The genus *Meligethes* (Coleoptera) was considered to be a flower herbivore rather than a pollinator, and infested flowers were discarded from the analysis. For each patch and day, only mature and fully opened flowers in the video frame were considered.

Pollinator service was summarized by the following variables. *Visitation rate* was the total number of insect-flower interactions detected per day (*abundance*) divided by the total number of open flowers visible in the video frame. *Pollination ratio* was the number of flowers visited at least once during the day to the total number of flowers in the video frame (analysed as fraction). *Pollinator richness* was the total number of different taxa/morphotypes observed, independent of flower number in the video frame. We also calculated the biodiversity/*Shannon index* (Shannon 1948) based on pollinator abundance and richness at the level of camera and day. The complete sample size was: 13 populations × 1–2 years of recording per population × 10–12 cameras per population and year × 2–4 days of recording per camera = 382 patches and days.

### Population and site characteristics

We quantified several characteristics of populations and patches related to the hypothesized mechanisms by which pollinator service may decline toward range limits. *Population size* was calculated based on the area of occurrence of *A. lyrata* multiplied by the average of local plant density. The area of occurrence [m^2^] was assessed by carefully screening for the presence and absence of the species with a global positioning tracker (GPS, Garmin, eTrex 20x, Olathe, Kansas, USA). Local plant density was the total number of plants per m^2^ at each patch where a camera was set up. *Local flower density* was the total number of open *A. lyrata* flowers per m^2^ at each patch. We assessed *flower size* on one flower of 40 randomly chosen mature plants in each population during midday when flowers were fully open. Flower size was the length of the ovary multiplied by the maximal width of the corolla [mm]. Finally, *plant species richness* was the total number of flowering plant species co-occurring temporally and spatially with flowering *A. lyrata*. To assess the effect of temperature, two data loggers (DS1922L, Maxim *iButton, San José,* CA, USA) collected air temperature hourly at each population while cameras were recording. In the analysis on their relationship with pollinator service, *population size*, mean *flower size*, *plant species richness*, and daily *mean temperature* were predictors on the level of the population, while *local flower density* was a predictor on the level of the patch monitored within population and year.

### Statistical analysis

Daily *visitation rate* and *pollination ratio* were the main dependent variables. To test whether pollinator service declined from the centre of the distribution toward the edges (research question I), we used generalised linear mixed-effects models, analysed with restricted maximum likelihood and the *bobyqa* optimizer, with the R packages lme4 (Bates et al. 2015) and lmerTest (Kuznetsova et al. 2017) in R (R Core Team 2019). Fixed effects were explored for their relevance by model selection, based on the Akaike information criterion, AIC: latitude (1); latitude and its square term (2); latitude and elevation (3); and latitude, its square term, and elevation (4), apart from the null model with only an intercept. Covariates were mean-centred (before taking the square; type 3-testing was deployed). Random effects were hierarchically structured and included camera in a population and year, and population (code provided in S1). Secondary dependent variables were *pollinator richness* and *Shannon index* for pollinators. Mechanistic variables were also tested for a relationship with latitude, its square term, and elevation by model selection. These included *population size* (log_10_-transformed), *local flower density* (log_10_-transformed), *flower size*, *plant species richness,* and *mean temperature*. Random effect was population (none for population size and plant species richness).

The mechanistic hypotheses about pollinator service were addressed by testing the effects of log_10_-transformed *population size*, log_10_-transformed *local flower density* and its square term, *flower size*, *plant species richness*, and *mean temperature* on the dependent variables of daily *visitation rate* and *pollination ratio* (research question II). Covariates were mean-centred (before taking the square), and type 3-testing was deployed. Random effects were camera within population and year, and population as well as heterogeneity in slopes on local flower density, its square term, and mean temperature on the day of observation within populations. Finally, we checked for variance inflation, and for residual autocorrelation as suggested by Diniz-Filho et al. (2003) by Moran’s I testing implemented in the package *ape* (Paradis and Schliep 2019).

## Results

**Fig. 2:**
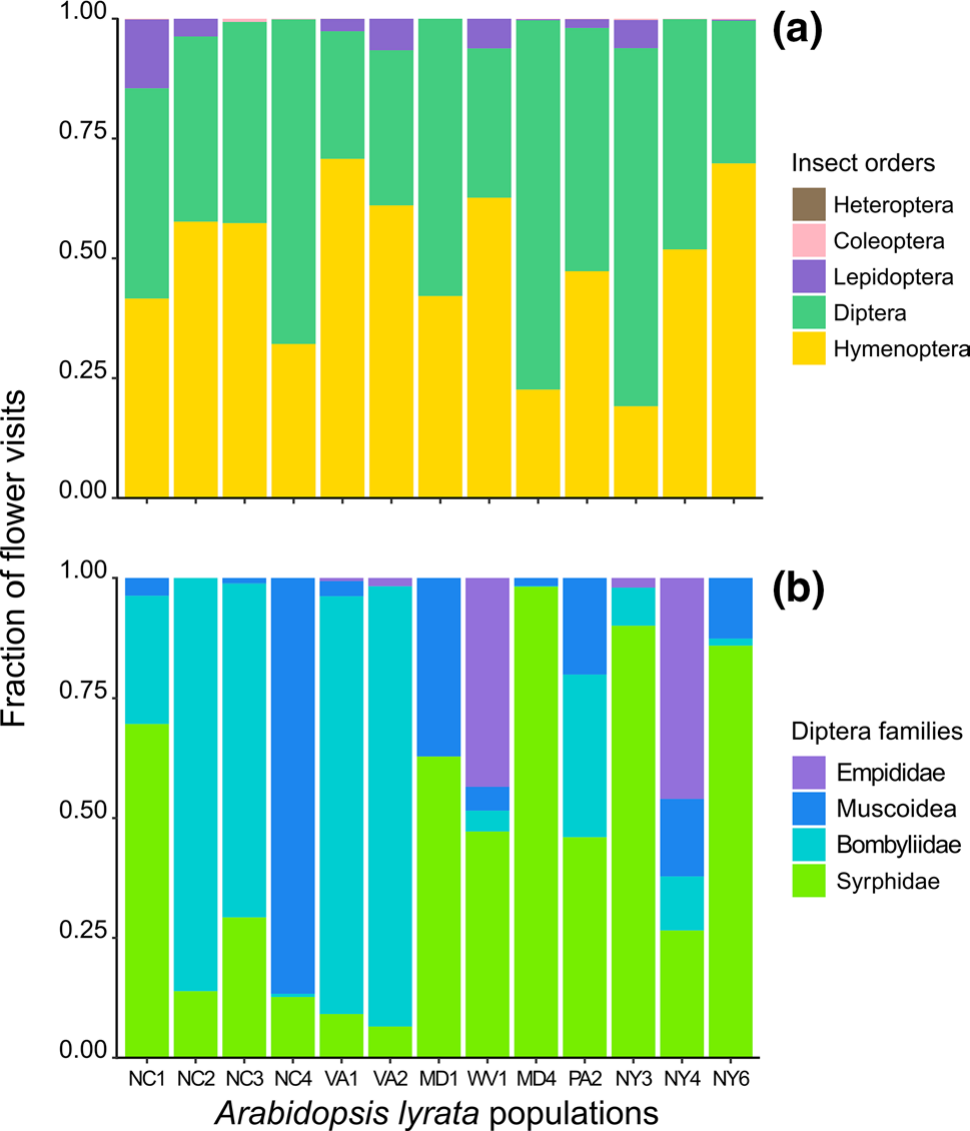
Fraction of flower visits by **a** different insect orders or **b** families within Diptera in 13 populations of *Arabidopsis lyrata*. Populations are sorted from south (left) to north (right). For population abbreviations see legend Fig. 1

The total observation effort across all populations, cameras, and days was 4522 h. During this time, 7310 *A. lyrata* flowers were monitored, and 17,508 insects visited them. Visitors fell into 67 morphotypes, and 88% were identified at the level of order (see Table S2 for the full list). The remaining 12% of visits were categorized as unidentifiable. About 49% of the insects were hymenopterans of the Apocrita group, followed by 48% dipterans, 3.2% lepidopterans, and 0.1% coleopterans (Table S3). The fraction of each insect order varied among populations, but there was no obvious trend with latitude (Fig. 2a). Within Diptera, Syrphidae and Bombyliidae were represented best (46% and 32%, respectively), followed by Muscoidea and Empididae (Fig. 2b; Table S4). While southern *A. lyrata* populations were visited more often by bombyliids, centre and northern populations were visited more frequently by syrphids (Fig. 2b). Some taxa were observed in more than one population, particularly the hoverfly *Toxomerus marginatus,* which was a common visitor in all populations. Although several other insects occurred across the entire latitudinal gradient, there were also unique pollinators in each population. Some of the pollinator service variables were correlated (Fig. S1A): *visitation rate* and *pollination ratio* (*r* = 0.51), and *pollinator* *richness* and *Shannon index* (*r* = 0.93).

The mechanistic variables hypothesized to be associated with pollinator service varied greatly. *Population size* ranged from 600 to 378,000 plants, and *local flower density* varied from 23 to 255 per m^2^ (Tables S5, S6). *Flower size* was largest in a mixed-mating population in Virginia (VA2, see Table S6). The *richness* of flowering *plant species* ranged from 0 to 7 species (Tables S5, S7). Several of the mechanistic factors were significantly correlated (Fig. S1B).

*I. Does pollinator service decline from the centre toward range edges?*

Model selection for pollinator service and mechanistic variables indicated that the model with latitude alone—not including its square term or elevation—was often among the best supported by the data, apart from the model including the intercept only (Tables 1, S8). Therefore, we got estimates for the model with latitude.

**Fig. 3:**
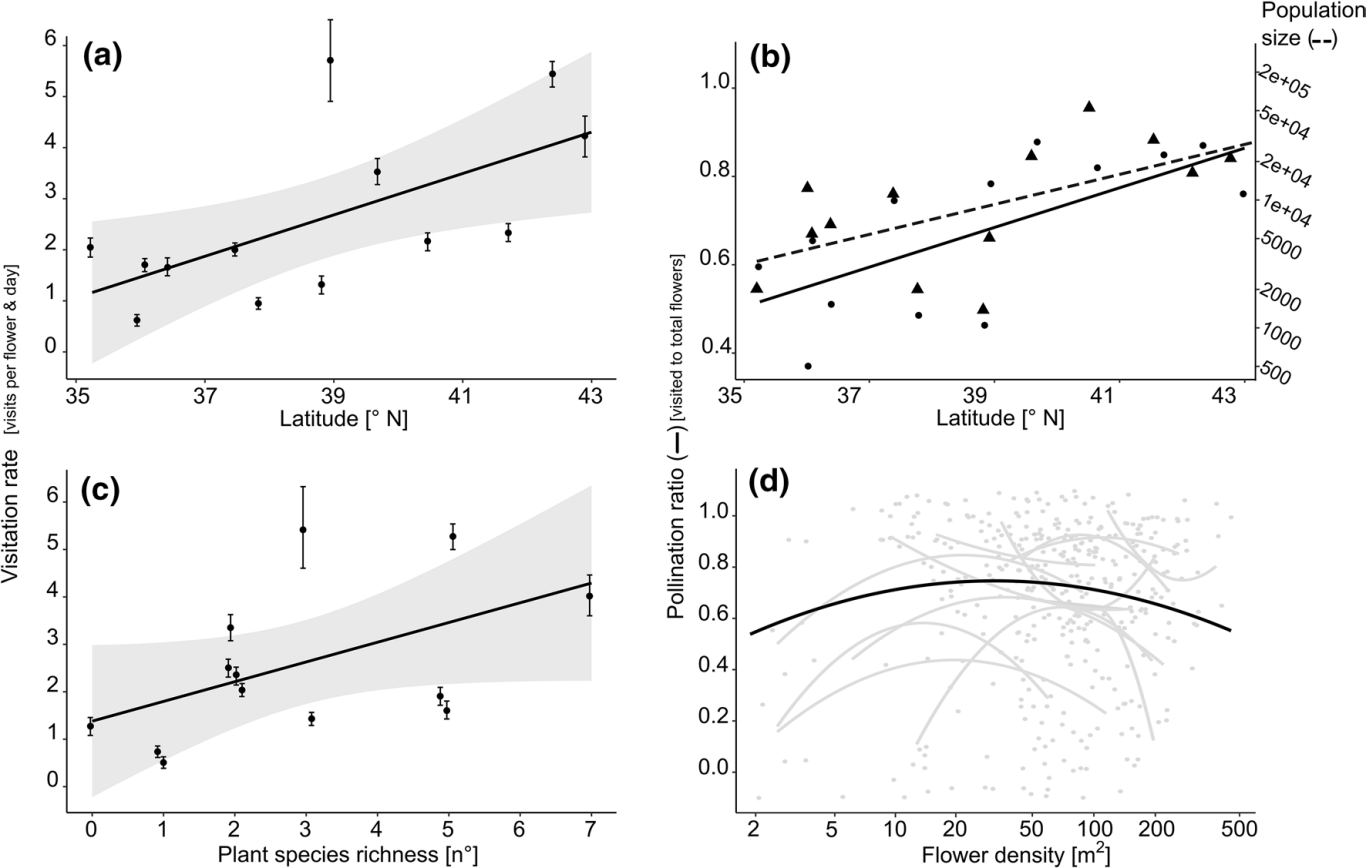
Relationship between **a** visitation rate (per flower and day) and latitude, **b** pollination ratio (visited flowers to total flowers) and latitude, **c** visitation rate and plant species richness, **d** pollination ratio and local flower density in *Arabidopsis lyrata* populations. In panels **a** and **c**, symbols represent population estimates, in panel d, they represent patch estimates. In **b**, circles represent pollination ratio and triangles population size. Population means were calculated by averaging first across replicate cameras within population and year, then across cameras, and finally across years, if applicable. In **a** and **c**, standard errors calculated on the highest level of averaging are indicated. The black lines are model-predicted relationships (dashed line for population size), with lower and upper 95% confidence intervals. In **d**, curves represent quadratic relationships between pollination ratio and log_10_-transformed local flower density, across populations (in black) and for each population separately (in grey). For statistics, see Tables 1 and 2

Both daily *visitation rate* and *pollination ratio* were positively correlated with latitude (Fig. 3a and b, Table 1). Southern populations such as NC2 and VA2 received on average less than one visit per day, and about 60% of flowers remained unvisited (Table S9). In contrast, in centre and northern populations such as WV1 and NY4, 3–5 pollinators per flower and day were observed and less than 20% of flowers remained unvisited. Of the five mechanistic environmental variables, only *population size* was associated with latitude, in a positive direction (Fig. 3b, Table 1).

*II. What are the mechanisms for reduced pollinator service?*

**Table 1:** Results of mixed-effects models testing for an association between latitude of *Arabidopsis lyrata* populations and (a) estimates of pollinator service or (b) mechanistic variables potentially affecting pollinators

		Latitude		*R*^2^ *m*	*R*^2^ *c*
		Estimate	SE		
(a) Pollinator service	*N*				
Visitation rate	382	0.412**	0.150	0.095	0.578
Pollination ratio	382	0.045**	0.015	0.134	0.505
Pollinator richness	382	0.091	0.134	0.014	0.568
Shannon index	382	0.024	0.034	0.013	0.491
(b) Mechanistic variables					
Population size (log_10_)	13	0.215*	0.081	0.369	0.369
Local flower density (log_10_)	166	−0.009	0.041	0.002	0.508
Flower size	520	0.073	0.802	0.000	0.668
Plant sp. richness	13	0.192	0.231	0.054	0.054
Mean *T*°	39	−0.361	0.386	0.053	0.611

The number of replicates (*N*) is the number of original observations: camera and day for pollinator data, or population (13), patch (166), flower measured (520) or day recording (39) for mechanistic variables. Regression coefficients (estimate) with standard error (SE) are reported. Significance is indicated: * *P* < 0.05, ** *P* < 0.01, *** *P* < 0.001. *R*^2^*m* and *R*^2^*c* stand for the marginal and the conditional coefficient of determination, respectively. Results for random effects are not shown

**Fig. 4:**
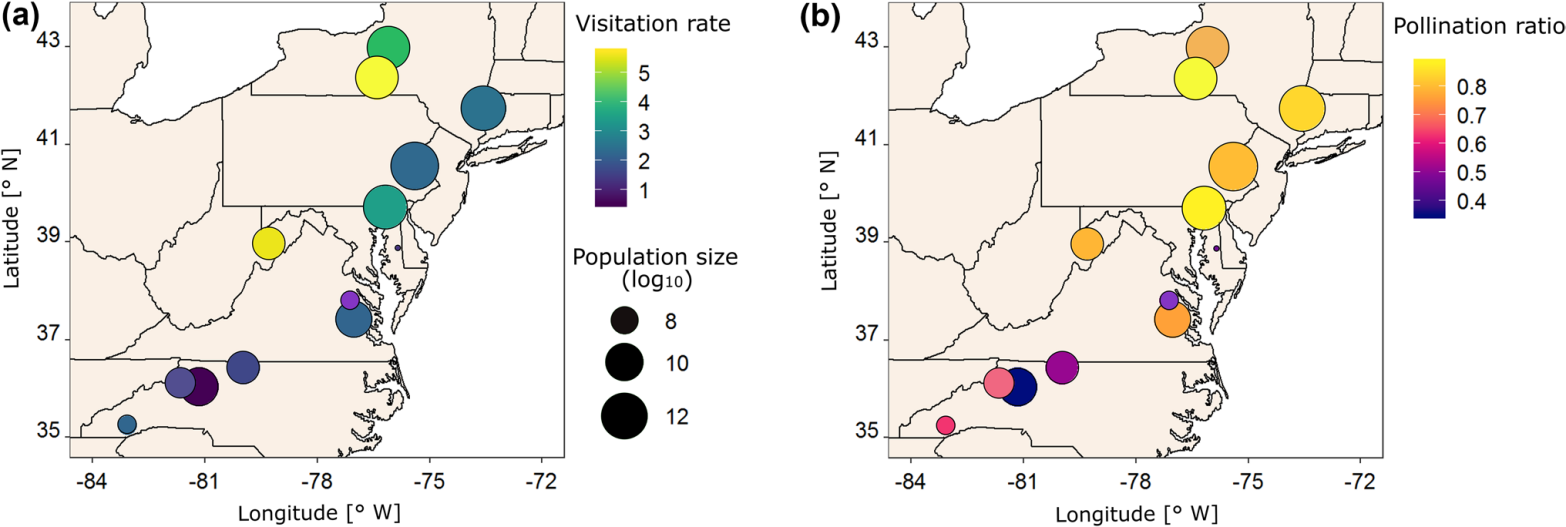
Maps illustrating the relationships among **a** visitation rate or **b** pollination ratio with population size, and the geography of the 13 populations of *Arabidopsis lyrata.* The size of the circles represents the population size, and the colour indicates the population mean of **a** visitation rate or **b** pollination ratio. Population means were calculated by averaging first across replicate cameras within population and year, then across cameras, and finally across years, if applicable

To address research question II, we tested for an association between pollinator service and potential mechanistic variables independent of range position (Table 2). *Pollination ratio*, and *visitation rate* as a trend, were positively related with *population size*. The result is illustrated in Fig. 4 on a map, with the large northern and centre populations having higher *visitation rates* and *pollination ratios*. There was also an increase in *visitation rate* with *plant species richness* (Fig. 3c). However, *visitation rate* and *pollination ratio* decreased with high *local flower density* of *A. lyrata*; local *flower density* was significantly negatively related with *visitation rate* and the square term of *local flower density* was significant and negative for *pollination ratio* (Table 2). The quadratic term implied further that also at low patch density of flowers, the chance of a flower being visited on a day was lower (Fig. 3d). The exact shape of curves depicting the relationship between *pollination ratio* and *local flower density* differed considerably among populations, together with their position along the gradient of local flower density (Fig. 3d). Nevertheless, the pattern of increasing and then decreasing pollination ratio along the flower density gradient was fairly robust across populations.

**Table 2:** Results of mixed-effects models testing for an association between population size, local flower density, flower size, flowering plant species richness, and daily mean temperature on pollinator service to *Arabidopsis lyrata* flowers

		Population size (log_10_)		Local flower density (log_10_)		Local flower density^2^		Flower size		Plant sp. richness		Mean T°		*R*^2^*m*	*R*^2^*c*
		Estimate	SE	Estimate	SE	Estimate	SE	Estimate	SE	Estimate	SE	Estimate	SE		
Pollinator service	*N*														
Visitation rate	382	0.845(*)	0.47	−1.776*	0.89	0.364	1.66	0.045	0.06	0.397*	0.19	0.042	0.06	0.13	0.65
Pollination ratio	382	0.141**	0.05	−0.088	0.06	−0.166**	0.06	0.001	0.01	0.013	0.02	0.004	0.01	0.14	0.56
Richness	382	0.231	0.34	0.836*	0.39	−0.257	0.40	0.090*	0.04	0.198	0.15	0.068	0.05	0.16	0.63
Shannon index	382	0.058	0.09	0.223*	0.11	−0.051	0.11	0.021(*)	0.01	0.051	0.04	0.015	0.01	0.13	0.56

Pollinator services are the dependent variables, while the mechanistic predictors are the independent variables. All predictors were mean-centred. Regression coefficients of fixed effects (estimate) with standard error (SE) are reported. Significance is indicated: (*) *P* < 0.1, * *P* < 0.05, ** *P* < 0.01, *** *P* < 0.001. R^2^m and R^2^c stand for the marginal and the conditional coefficient of determination, respectively. The *bobyqa* optimizer was used

Analyses on *pollinator richness* and *Shannon index* revealed a positive role of *local flower density* (Table 2). Both estimates of pollinator diversity increased with increasing flower density. Furthermore, larger flowers attracted a more diverse community of pollinators; the pattern was significant for *pollinator richness* and a trend for the *Shannon index*. However, when both dependent variables were corrected for flower numbers in the frame of the camera, these effects were not found. Finally, *mean temperature* was not related with any of the four estimates of pollinator service.

Despite some correlation structure among independent variables, variance inflation factors were all < 2 (Table S10). Furthermore, Moran’s *I* on residuals of the four models were not significantly different from 0 (all *P* > 0.4).

## Discussion

Our study provides evidence that factors acting locally or on the scale of populations and regions are associated with variation in pollinator service across a plant’s geographic distribution, with important factors being density and population size. On a local scale, pollinator service—the chance of an *A. lyrata* flower being visited at least once in a day—was reduced if the flower density was too low or too high (Fig. 3d). On a geographic scale, populations in the south, that were significantly smaller, had lower pollination ratios, several with fewer than half of the flowers being visited on a day (Fig. 4b). This latter pattern could help establish the southern range limit of *A. lyrata*. Below we discuss these and other results in the context of species’ range limits and pollination biology more generally.

*I. Does pollinator service decline from the centre toward range edges?*

Ecological niche modelling on climate data showed that range limits of *A. lyrata* in the south and north reflect niche limits (Lee-Yaw et al. 2018). A similar conclusion was supported by a transplant experiment to sites beyond the species’ range in south and north, which showed that the southern—but not the northern range limit—reflects niche limits (Sánchez-Castro et al. 2021 unpublished data). The main causes of performance decline at southern sites were climatic. The results found here add that also pollinator service is not favourable in *A. lyrata* populations at the southern range limit (Table 1, Fig. 3). Populations in the south were small, and in the smallest population, flowers had an approximately 50% chance of receiving no pollinator visit in a day, compared with the lower than 20% chance of no visitation in the largest population (model predictions, Fig. 3b). To evaluate the likely biological impact of this result, a couple of additional factors need to be considered. On the one hand, flowers are generally receptive to pollinators for a short time, but typically for longer than a day, which increases the chance of being visited at least once by a pollinator compared to our numbers. On the other hand, our field observations were collected under optimal conditions, when the weather was ideal for insect pollinators. Therefore, we think that across an entire reproductive season, many flowers in small southern populations may suffer from low insect visitation. Even if pollinators are not a primary source causing range limits, chronically low pollinator service may nevertheless contribute to reduced reproduction and small population size (Groom 1998). In contrast to the south, northern range-edge populations did not receive reduced pollinator service. These results, in combination with those of the transplant experiment described earlier, suggest that northern edge populations are limited neither by climate nor by a lack of pollinator service whereas southern populations are.

Previous studies have indicated that pollinators may enforce range limits. For example, populations of *Witheringia solanacea* in Costa Rica had greater visitation and fruit set in a lower montane site than at the upper elevational limit (Stone and Jenkins 2008). Similar results were found for *Embothrium coccineum* in northwestern Patagonia, where lower pollinator service occurred in populations at the eastern range limit, and climatic variables such as precipitation were not more important than biotic interactions (Chalcoff et al. 2012). For *Clarkia xantiana* in the Sierra Nevada, the abundance and visitation rates of pollinators decreased and pollen limitation increased at the range limits compared to centre populations (Moeller et al. 2012). However, Hargreaves et al. (2015) found no evidence that pollination activity decreased at the upper range limit for *Rhinanthus minor* in the Rocky Mountains. These mixed results motivated our examination of mechanisms that may affect pollinator service and whether they vary across the latitudinal gradient.

*II. What are the mechanisms for reduced pollinator service?*

One of the four hypothesized mechanisms for reduced pollinator service was supported at the southern range edge (Table 2). Southern *A. lyrata* populations were smaller, and these small populations attracted fewer insect pollinators (Tables 1 and 2, Fig. 3b). The positive relationship between population size and pollination ratio suggests the potential for an Allee effect in pollination. Courchamp et al. (1990) defined the Allee effect as “… a scenario in which populations at low numbers are affected by a positive relationship between population growth rate and density …”. In our study, we did not assess the downstream effect of reduced pollination ratio on reproductive success and population growth rate. However, e.g. the now classic study performed by Groom (1998) suggests that this link is likely. Groom showed in experimental populations of *Clarkia concinna* that flowers of small and isolated populations were visited less frequently by pollinators than those of large populations—based on pollen counts. Furthermore, plants in small and isolated populations had a lower seed set, which is a vital rate determining plant population growth.

Our study found also the potential for an Allee effect in pollination independent of range position, on a local scale, within populations. The relationship between pollination ratio and density was hump-shaped, with the highest chance of a flower being visited in a day occurring at intermediate flower density (Fig. 4d). In other words, density dependence of pollination ratio was positive at low densities, whereas it was negative at high densities. For the number of visits per flower and day, only negative density dependence was supported. While some previous studies on pollination also revealed positive density dependence (Kunin 1997; Delmas et al. 2016; Nielsen and Ims 2000), there were also some showing a negative correlation (Hendrickson et al. 2018; Grindeland et al. 2005;) or no relationship (Kirchner et al. 2005). Our study may provide some insights why mixed results may occur. First, we found variation in the relationship between pollinator service and density depending on populations, mainly because they differed in the range they covered on the density gradient (Fig. 4d). Second, differences in results may occur depending on how pollinator service is quantified. We found the hump-shaped pattern with density for pollination ratio, which emphasizes the chance of reproduction, but not visitation rate, that may give too much emphasis on some flowers being visited frequently.

Independent of range position, we found that visitation rate of flowers increased with plant species richness (Table 2). This result is well in line with research that showed that the diversity of floral resources increases the visitation rate (Ghazoul 2006; Hegland and Boeke 2006) and that it attracts a greater number of pollinator species (Lázaro and Totland 2010). In turn, pollinator diversity was significantly increased by the local density of *A. lyrata* (Table 2). As a side result, we did not find that the one selfing population (NC1) had small flowers; in fact, the one mixed-mating population (VA2) had the largest flowers (Table S6). Therefore, our results do not align with the idea of a reduction in flower size when there is a shift in the mating system from outcrossing to selfing. However, most self-compatible populations require insect pollinators for pollen deposition on stigmas. Finally, our daily mean temperature data did not show a correlation with latitude or pollinator service (Tables 1 and Table 2). This is probably because the recording of pollinators occurred between 20 and 30 °C at all sites (Table S5).

*III. Pollination biology of A. lyrata*

A recent study on one *A. lyrata* population on Isle Royale pointed to syrphids as main flower visitors, in particular the genus *Toxomerus* (Edwards et al. 2019). By extending the geographical scope, we found that both Hymenoptera and Diptera were equally important as main pollinators, while Lepidoptera represented a small proportion of the visits (Fig. 2a). Within the Diptera, hoverflies were the most frequent family in the centre and northern populations, supporting the previous results of Edwards et al. (2019), while Bombyliidae dominated at lower latitudes (Fig. 2b). Even though we found some common pollinators in all populations such as *Toxomerus*, all populations and many flowers within populations were visited by multiple insect taxa. Results demonstrate that the pollination system is generalist that provides ecological flexibility in terms of reproduction for the plant and increases diversity in food resources for the pollinators (Waser et al. 1996; Fenster et al. 2004).

Furthermore, our research provided some noteworthy results on the distribution of pollinator diversity. First, we did not find that pollinator diversity was increased at southern compared to northern latitudes, as e.g. suggested by Schemske et al. (2009). However, despite population size of *A. lyrata* being lower in the south, and visitation rate and pollination ratio declining accordingly, pollinator diversity was not significantly lower. Second, pollinator diversity was higher on patches with a higher density of flowers, which could have been influenced by a sampling effect.

## Conclusion

Pollinator service was found to vary considerably across the distribution of *A. lyrata*. Southern range-edge populations had lower visitation by pollinators, and this was linked with their smaller population size. The result points to limited pollinator service as a stabilizer of range limits. Apart from this potential for an Allee effect in pollination on the level of the population, we also found evidence for the same effect on the level of local patches within populations. In patches of low density, the chance of a flower being visited at least once a day was lower compared to flowers of mid-density patches; at higher densities, density dependence changed to negative. The two levels of positive density dependence, under small population size and low local density, support the importance of Allee effects in pollination.

## Supplementary Information

Below is the link to the electronic supplementary material.

Supplementary file1 (DOCX 1242 kb)

## Data Availability

The datasets used and/or analysed during the current study are available from the corresponding author on reasonable request.
