# Supplementary material for: Reduced pollinator service in small populations of Arabidopsis lyrata at its southern range limit
Source: Oecologia. 2022 Sep 2;200(1-2):107–17. doi: 10.1007/s00442-022-05237-1 (PMC9547784; doi:10.1007/s00442-022-05237-1)
Supplement: Supplementary file 1 — Supplementary file1 (DOCX 1242 kb) [file 442_2022_5237_MOESM1_ESM.docx]

**Supporting information**

**Table S1** General information on the *Arabidopsis lyrata* populations used for this study. Populations are sorted by latitude. The following information is given: population abbreviation, name of location, position within the range, latitude, longitude, elevation, surface area with occurrences, the number of days of recording pollinators, the number of patches in the population, and the total number of hours of recording.

| Pop | Location | Origin | Latitude | Longitude | Elevation  [m a.s.l.] | Area [m^2^] | Days of recording | N° of patches | Total hours |
| --- | --- | --- | --- | --- | --- | --- | --- | --- | --- |
|  |  |  | [° N] | [° W] |  |  |  |  |  |
| NC1 | Tuckasegee | South | 35.25 | 83.08 | 1,015 | 184 | 3.5 | 21 | 379 |
| NC2 | Moravian Falls | South | 36.04 | 81.16 | 680 | 5,031 | 4 | 20 | 331 |
| NC3 | Blowing Rock | South | 36.11 | 81.66 | 1,108 | 682 | 5.5 | 6 | 400 |
| NC4 | Mayodan | South | 36.41 | 79.96 | 224 | 1,149 | 3 | 9 | 308 |
| VA1 | Sandybottom | South | 37.42 | 77.02 | 5 | 2,175 | 3 | 12 | 428 |
| VA2 | Aylett | South | 37.81 | 77.12 | 10 | 110 | 3 | 8 | 260 |
| MD1 | Martinak St. Park | Centre | 38.86 | 75.84 | 2 | 64 | 2 | 10 | 208 |
| WV1 | Hopeville | Centre | 38.96 | 79.29 | 395 | 1,001 | 3.5 | 10 | 307 |
| MD4 | Conowingo | Centre | 39.70 | 76.19 | 79 | 5,925 | 2 | 10 | 262 |
| PA2 | Allentown | Centre | 40.57 | 75.4 | 125 | 8,743 | 3 | 12 | 400 |
| NY3 | Dover Plains | North | 41.73 | 73.56 | 143 | 10,264 | 3 | 12 | 397 |
| NY4 | Ithaca | North | 42.35 | 76.39 | 442 | 3,222 | 3 | 12 | 424 |
| NY6 | Clark State Park | North | 43.00 | 76.09 | 229 | 7,185 | 4 | 20 | 418 |

**Table S2** List of insect species/morphotypes observed pollinating *Arabidopsis lyrata* sub. *lyrata* in the 13 populations studied (0 = absent; 1 = present)

| Population of *A. lyrata* | | | | | | | | | | | | | |
| --- | --- | --- | --- | --- | --- | --- | --- | --- | --- | --- | --- | --- | --- |
| Species/Morphotype | NC1 | NC2 | NC3 | NC4 | VA1 | VA2 | MD1 | WV1 | MD4 | PA2 | NY3 | NY4 | NY6 |
| *Alipia octomaculata* | 0 | 0 | 0 | 0 | 0 | 0 | 0 | 0 | 0 | 1 | 0 | 0 | 0 |
| *Anthocharis midea* | 1 | 1 | 0 | 0 | 0 | 0 | 0 | 0 | 0 | 0 | 0 | 0 | 0 |
| Apocrita morphotype 1 | 0 | 0 | 1 | 1 | 0 | 0 | 0 | 1 | 0 | 0 | 0 | 1 | 1 |
| Apocrita morphotype 2 | 0 | 1 | 1 | 0 | 1 | 0 | 1 | 0 | 1 | 0 | 0 | 1 | 0 |
| Apocrita morphotype 3 | 1 | 0 | 0 | 0 | 0 | 0 | 0 | 0 | 0 | 1 | 0 | 0 | 0 |
| Apocrita morphotype 4 | 0 | 0 | 0 | 0 | 0 | 0 | 0 | 0 | 1 | 1 | 0 | 1 | 0 |
| Apocrita morphotype 5 | 0 | 1 | 0 | 0 | 0 | 0 | 0 | 0 | 1 | 0 | 0 | 0 | 0 |
| Apocrita morphotype 6 | 0 | 0 | 0 | 0 | 1 | 1 | 0 | 1 | 0 | 0 | 0 | 0 | 0 |
| Apocrita morphotype 7 | 0 | 0 | 0 | 0 | 1 | 1 | 0 | 0 | 0 | 0 | 0 | 0 | 0 |
| Apocrita morphotype 8 | 0 | 0 | 0 | 0 | 1 | 0 | 0 | 0 | 0 | 1 | 0 | 0 | 0 |
| Apocrita morphotype 9 | 1 | 0 | 0 | 0 | 0 | 0 | 0 | 0 | 0 | 0 | 0 | 0 | 0 |
| Apocrita morphotype 10 | 0 | 0 | 0 | 0 | 0 | 0 | 0 | 0 | 0 | 0 | 1 | 0 | 0 |
| Apocrita morphotype 11 | 0 | 0 | 0 | 0 | 0 | 1 | 0 | 0 | 0 | 0 | 0 | 0 | 0 |
| Apocrita morphotype 12 | 0 | 0 | 0 | 0 | 0 | 0 | 0 | 0 | 0 | 0 | 0 | 1 | 0 |
| Apocrita morphotype 13 | 0 | 0 | 0 | 0 | 0 | 0 | 0 | 1 | 0 | 0 | 0 | 0 | 0 |
| Apocrita morphotype 14 | 0 | 0 | 1 | 0 | 0 | 0 | 0 | 0 | 0 | 0 | 0 | 0 | 0 |
| Apocrita morphotype 15 | 1 | 0 | 0 | 0 | 0 | 0 | 0 | 0 | 0 | 0 | 0 | 0 | 0 |
| *Asterocampa sp.* | 1 | 1 | 0 | 0 | 1 | 1 | 0 | 1 | 0 | 0 | 0 | 0 | 0 |
| *Bombus sp.* | 0 | 0 | 1 | 0 | 1 | 0 | 0 | 0 | 0 | 0 | 0 | 0 | 0 |
| *Bombylius major* | 1 | 1 | 1 | 1 | 1 | 1 | 0 | 0 | 1 | 1 | 1 | 1 | 1 |
| *Bombylius pulchellus* | 0 | 0 | 0 | 0 | 0 | 0 | 0 | 0 | 0 | 1 | 0 | 0 | 0 |
| *Bombylius pygmaeus* | 0 | 0 | 0 | 0 | 0 | 0 | 0 | 0 | 0 | 0 | 0 | 1 | 0 |
| *Callophrys grynaeus* | 0 | 0 | 0 | 0 | 0 | 0 | 0 | 0 | 0 | 1 | 1 | 0 | 0 |
| Chloropidae | 0 | 0 | 0 | 0 | 0 | 0 | 0 | 0 | 0 | 0 | 0 | 0 | 1 |
| *Chrysotoxum sp.* | 1 | 0 | 0 | 0 | 0 | 0 | 0 | 0 | 0 | 0 | 0 | 0 | 0 |
| Coleoptera | 0 | 0 | 0 | 0 | 0 | 0 | 0 | 0 | 0 | 0 | 0 | 0 | 1 |
| Conopidae | 0 | 0 | 0 | 0 | 0 | 0 | 0 | 0 | 0 | 0 | 0 | 1 | 0 |
| *Cupido comyntas* | 0 | 0 | 0 | 0 | 1 | 1 | 0 | 0 | 0 | 0 | 0 | 0 | 0 |
| Empididae | 0 | 0 | 0 | 0 | 0 | 0 | 0 | 0 | 0 | 0 | 0 | 1 | 0 |
| *Empis* morphotype 1 | 0 | 0 | 0 | 0 | 0 | 0 | 0 | 0 | 0 | 0 | 1 | 1 | 0 |
| *Empis* morphotype 2 | 0 | 0 | 0 | 0 | 0 | 0 | 0 | 1 | 0 | 0 | 0 | 0 | 0 |
| *Empis* morphotype 3 | 0 | 0 | 1 | 0 | 0 | 1 | 0 | 0 | 0 | 0 | 0 | 0 | 0 |
| *Epalpus* morphotype 1 | 0 | 0 | 0 | 0 | 0 | 0 | 0 | 0 | 0 | 0 | 0 | 1 | 0 |
| *Epalpus* morphotype 2 | 0 | 0 | 0 | 0 | 0 | 0 | 1 | 0 | 0 | 0 | 0 | 0 | 0 |
| *Eristalis saxorum* | 0 | 0 | 0 | 0 | 1 | 0 | 0 | 0 | 0 | 1 | 0 | 0 | 0 |
| *Eupeodes sp.* | 0 | 0 | 0 | 0 | 0 | 0 | 0 | 1 | 0 | 0 | 0 | 0 | 0 |
| *Eurythmia sp.* | 0 | 0 | 0 | 0 | 0 | 0 | 0 | 1 | 0 | 0 | 0 | 0 | 0 |
| Halictidae fam | 1 | 1 | 1 | 1 | 1 | 1 | 0 | 0 | 1 | 0 | 0 | 1 | 1 |
| *Heliophilus fasciatus* | 0 | 0 | 0 | 0 | 0 | 0 | 0 | 0 | 0 | 0 | 1 | 0 | 0 |
| *Hemipenthes sp.* | 0 | 0 | 0 | 0 | 0 | 0 | 0 | 1 | 0 | 0 | 0 | 0 | 0 |
| Population of *A. lyrata* | | | | | | | | | | | | | |
| Species/Morphotype | NC1 | NC2 | NC3 | NC4 | VA1 | VA2 | MD1 | WV1 | MD4 | PA2 | NY3 | NY4 | NY6 |
| Heteroptera | 0 | 0 | 0 | 0 | 1 | 0 | 0 | 0 | 0 | 0 | 0 | 0 | 0 |
| *Mallota bautias* | 0 | 0 | 0 | 0 | 0 | 0 | 0 | 0 | 0 | 1 | 0 | 0 | 0 |
| *Mesembrina sp.* | 1 | 0 | 1 | 0 | 0 | 0 | 0 | 0 | 1 | 0 | 0 | 1 | 1 |
| Moth sp.1 | 1 | 0 | 0 | 0 | 0 | 0 | 0 | 0 | 0 | 0 | 0 | 0 | 0 |
| Moth sp.2 | 0 | 0 | 0 | 0 | 0 | 0 | 0 | 0 | 0 | 0 | 1 | 0 | 0 |
| Moth sp.3 | 0 | 0 | 0 | 0 | 0 | 0 | 0 | 0 | 0 | 0 | 0 | 0 | 1 |
| Moth sp.4 | 0 | 0 | 0 | 0 | 0 | 0 | 0 | 0 | 0 | 0 | 0 | 0 | 1 |
| Muscoidea sp.1 | 0 | 0 | 0 | 0 | 0 | 0 | 0 | 0 | 0 | 1 | 0 | 0 | 0 |
| Muscoidea sp.2 | 1 | 0 | 0 | 0 | 1 | 0 | 0 | 0 | 0 | 0 | 1 | 1 | 1 |
| Muscoidea sp.3 | 1 | 0 | 0 | 1 | 0 | 0 | 0 | 1 | 1 | 0 | 0 | 0 | 0 |
| Nomada sp.1 | 0 | 0 | 0 | 0 | 0 | 0 | 0 | 0 | 1 | 0 | 0 | 1 | 1 |
| Nomada sp.2 | 0 | 0 | 0 | 0 | 0 | 0 | 0 | 0 | 0 | 0 | 1 | 0 | 0 |
| *Phyciodes sp.* | 1 | 0 | 0 | 0 | 1 | 0 | 0 | 0 | 0 | 0 | 0 | 0 | 0 |
| *Platycheirus sp.* | 1 | 0 | 1 | 1 | 0 | 0 | 0 | 0 | 0 | 1 | 1 | 0 | 0 |
| *Pyrausta orphisalis* | 1 | 0 | 0 | 0 | 0 | 0 | 0 | 0 | 0 | 0 | 0 | 0 | 0 |
| *Sciomyzidae sp.* | 0 | 0 | 0 | 0 | 1 | 0 | 0 | 0 | 0 | 0 | 0 | 0 | 0 |
| *Sericomyia sp.* | 0 | 0 | 0 | 0 | 0 | 0 | 0 | 0 | 0 | 1 | 1 | 0 | 0 |
| *Sphaerophoria sp.* | 0 | 0 | 0 | 0 | 0 | 0 | 0 | 1 | 1 | 0 | 0 | 1 | 0 |
| Syrphidae sp.1 | 1 | 0 | 0 | 0 | 0 | 0 | 0 | 1 | 0 | 0 | 0 | 0 | 0 |
| Syrphidae sp.2 | 0 | 0 | 0 | 0 | 0 | 0 | 0 | 0 | 0 | 0 | 0 | 0 | 1 |
| Syrphidae sp.3 | 0 | 0 | 0 | 0 | 0 | 0 | 0 | 1 | 0 | 0 | 0 | 0 | 0 |
| Syrphidae sp.4 | 0 | 0 | 0 | 0 | 0 | 0 | 0 | 0 | 0 | 0 | 0 | 1 | 0 |
| *Syrphus sp.* | 1 | 0 | 0 | 0 | 0 | 0 | 0 | 0 | 0 | 0 | 0 | 0 | 0 |
| *Thyris sepulchralis* | 0 | 0 | 0 | 0 | 1 | 0 | 0 | 0 | 0 | 0 | 0 | 0 | 0 |
| *Toxomerus germinatus* | 0 | 0 | 0 | 0 | 0 | 0 | 0 | 0 | 0 | 0 | 1 | 0 | 0 |
| *Villa fumicosta* | 0 | 0 | 0 | 0 | 0 | 0 | 0 | 0 | 0 | 1 | 0 | 0 | 0 |

**Table S3** Diversity in insect orders visiting *A. lyrata* populations depicted by their fraction of flower visits, in percent

| Fraction of visits by the main insect orders [%] | | | | | | | | |
| --- | --- | --- | --- | --- | --- | --- | --- | --- |
| Population |  | Hymenoptera |  | Diptera |  | Lepidoptera |  | Coleoptera |
| NC1 |  | 41.6 |  | 43.9 |  | 14.4 |  | 0.2 |
| NC2 |  | 57.7 |  | 38.6 |  | 3.7 |  | 0.1 |
| NC3 |  | 57.4 |  | 42.0 |  | 0.0 |  | 0.7 |
| NC4 |  | 32.1 |  | 67.7 |  | 0.0 |  | 0.0 |
| VA1 |  | 70.8 |  | 26.6 |  | 2.6 |  | 0.0 |
| VA2 |  | 61.1 |  | 32.4 |  | 6.6 |  | 0.0 |
| MD1 |  | 42.1 |  | 57.9 |  | 0.0 |  | 0.0 |
| WV1 |  | 62.7 |  | 31.1 |  | 6.2 |  | 0.0 |
| MD4 |  | 22.6 |  | 77.0 |  | 0.2 |  | 0.1 |
| PA2 |  | 47.3 |  | 50.8 |  | 1.8 |  | 0.1 |
| NY3 |  | 19.2 |  | 74.7 |  | 5.9 |  | 0.3 |
| NY4 |  | 51.9 |  | 48.0 |  | 0.0 |  | 0.1 |
| NY6 |  | 69.8 |  | 29.8 |  | 0.2 |  | 0.2 |
|  |  |  |  |  |  |  |  |  |
| Average |  | 48.9 |  | 47.7 |  | 3.2 |  | 0.1 |

**Table S4** Diversity in Diptera families visiting *A. lyrata* populations depicted by their fraction of flower visits, in percent

| Fraction of visits by main Diptera families [%] | | | | | | | | |
| --- | --- | --- | --- | --- | --- | --- | --- | --- |
| Population |  | Syrphidae |  | Bombyliidae |  | Muscoidea |  | Empididae |
| NC1 |  | 69.6 |  | 26.8 |  | 3.6 |  | 0.0 |
| NC2 |  | 13.9 |  | 86.1 |  | 0.0 |  | 0.0 |
| NC3 |  | 29.3 |  | 69.6 |  | 1.1 |  | 0.0 |
| NC4 |  | 12.7 |  | 0.7 |  | 86.7 |  | 0.0 |
| VA1 |  | 9.1 |  | 87.2 |  | 3.0 |  | 0.7 |
| VA2 |  | 6.5 |  | 91.9 |  | 0.0 |  | 1.6 |
| MD1 |  | 62.9 |  | 0.0 |  | 37.1 |  | 0.0 |
| WV1 |  | 47.2 |  | 4.3 |  | 5.0 |  | 43.5 |
| MD4 |  | 98.3 |  | 0.1 |  | 1.6 |  | 0.0 |
| PA2 |  | 46.0 |  | 33.9 |  | 20.1 |  | 0.0 |
| NY3 |  | 90.1 |  | 7.9 |  | 0.0 |  | 1.9 |
| NY4 |  | 26.6 |  | 11.3 |  | 16.2 |  | 46.0 |
| NY6 |  | 86.0 |  | 1.5 |  | 12.5 |  | 0.0 |
|  |  |  |  |  |  |  |  |  |
| Average |  | 46.0 |  | 32.4 |  | 14.4 |  | 7.2 |

**Table S5** Data on population size, total flower census size, flowering plant species richness, and temperature conditions for the 13 *Arabidopsis lyrata* populations studied. The table lists plant population size and flower census size, calculated based on the mean of density counts on 12 independent 1m^2^ plots and the total area where the population occurred. The table also shows averages of daily maximum, mean, and minimum temperatures across the days while cameras recorded.

| Population |  | Population size x10^3^ |  | Flower census size x10^3^ |  | Plant species richness |  | Max T° [°C] |  | Mean T° [°C] |  | Min T° [°C] |
| --- | --- | --- | --- | --- | --- | --- | --- | --- | --- | --- | --- | --- |
|  |  |  |  |  |  |  |  |  |  |  |  |  |
| NC1 |  | 1.1 |  | 11.8 |  | 5 |  | 24.0 |  | 19.3 |  | 12.8 |
| NC2 |  | 28.7 |  | 253.1 |  | 1 |  | 29.5 |  | 26.4 |  | 23.0 |
| NC3 |  | 5.8 |  | 63.3 |  | 3 |  | 22.1 |  | 17.3 |  | 11.9 |
| NC4 |  | 9 |  | 88.7 |  | 5 |  | 36.2 |  | 29.2 |  | 18.2 |
| VA1 |  | 18.3 |  | 254.7 |  | 2 |  | 26.1 |  | 22.5 |  | 16.2 |
| VA2 |  | 1.1 |  | 13.3 |  | 1 |  | 27.5 |  | 22.4 |  | 13.5 |
| MD1 |  | 0.6 |  | 1.4 |  | 0 |  | 23.7 |  | 21.7 |  | 19.0 |
| WV1 |  | 7.6 |  | 22.8 |  | 3 |  | 30.0 |  | 25.5 |  | 17.8 |
| MD4 |  | 91.2 |  | 760.2 |  | 2 |  | 25.7 |  | 22.4 |  | 17.9 |
| PA2 |  | 378.1 |  | 2226.6 |  | 2 |  | 26.7 |  | 20.8 |  | 14.8 |
| NY3 |  | 135.1 |  | 612.4 |  | 2 |  | 23.8 |  | 18.9 |  | 12.1 |
| NY4 |  | 65.5 |  | 303.4 |  | 5 |  | 21.8 |  | 16.9 |  | 11.0 |
| NY6 |  | 74.9 |  | 225.1 |  | 7 |  | 26.9 |  | 23.5 |  | 16.8 |

**Table S6** Data on flower size, plant density, and local flower density for the 13 *Arabidopsis lyrata* populations studied. The table lists population means and standard errors of corolla width, ovary length and flower size based on 40 replicate flowers per population. Plant and local flower density were calculated based on the mean of density counts on 12 plots of 1m^2^ with *A. lyrata* occurrence in each population.

| Pop |  | Corolla width [mm] | |  | Ovary length [mm] | |  | Flower size  [mm^2^] | |  | Local plant density [m^2^] | |  | Local flower density [m^2^] | |
| --- | --- | --- | --- | --- | --- | --- | --- | --- | --- | --- | --- | --- | --- | --- | --- |
|  |  | Mean | SE |  | Mean | SE |  | Mean | SE |  | Mean | SE |  | Mean | SE |
| NC1 |  | 8.57 | 0.17 |  | 3.47 | 0.06 |  | 30.04 | 1.00 |  | 6.14 | 1.03 |  | 64.24 | 12.11 |
| NC2 |  | 9.16 | 0.13 |  | 3.62 | 0.05 |  | 33.34 | 0.89 |  | 5.70 | 1.03 |  | 50.30 | 5.73 |
| NC3 |  | 8.83 | 0.11 |  | 3.33 | 0.05 |  | 29.54 | 0.72 |  | 8.50 | 2.14 |  | 92.75 | 12.94 |
| NC4 |  | 8.11 | 0.12 |  | 3.39 | 0.06 |  | 27.66 | 0.81 |  | 7.80 | 1.50 |  | 77.20 | 18.41 |
| VA1 |  | 11.35 | 0.14 |  | 4.32 | 0.05 |  | 49.05 | 0.85 |  | 8.42 | 1.22 |  | 117.08 | 16.94 |
| VA2 |  | 10.48 | 0.13 |  | 4.06 | 0.05 |  | 42.66 | 0.91 |  | 10.12 | 2.87 |  | 120.88 | 17.42 |
| MD1 |  | 8.05 | 0.12 |  | 3.26 | 0.05 |  | 26.34 | 0.68 |  | 9.00 | 2.36 |  | 22.50 | 5.02 |
| WV1 |  | 8.01 | 0.21 |  | 3.39 | 0.04 |  | 27.33 | 0.93 |  | 7.60 | 2.43 |  | 22.80 | 6.39 |
| MD4 |  | 8.56 | 0.12 |  | 3.23 | 0.05 |  | 27.79 | 0.69 |  | 15.40 | 3.01 |  | 128.30 | 26.96 |
| PA2 |  | 8.69 | 0.12 |  | 3.35 | 0.04 |  | 29.25 | 0.67 |  | 43.25 | 7.97 |  | 254.67 | 27.18 |
| NY3 |  | 9.94 | 0.08 |  | 3.58 | 0.03 |  | 35.68 | 0.51 |  | 13.17 | 4.51 |  | 59.67 | 8.44 |
| NY4 |  | 10.54 | 0.15 |  | 3.62 | 0.03 |  | 38.32 | 0.79 |  | 20.33 | 3.90 |  | 94.17 | 17.07 |
| NY6 |  | 9.02 | 0.13 |  | 3.44 | 0.03 |  | 31.07 | 0.65 |  | 10.43 | 1.20 |  | 31.33 | 5.71 |

**Table S7** List of flowering plant species at each population (0 = absent; 1 = present)

|  | Population of A. lyrata | | | | | | | | | | | | |
| --- | --- | --- | --- | --- | --- | --- | --- | --- | --- | --- | --- | --- | --- |
| Plant species | NC1 | NC2 | NC3 | NC4 | VA1 | VA2 | MD1 | WV1 | MD4 | PA2 | NY3 | NY4 | NY6 |
| *Alliaria petiolata* | 0 | 0 | 0 | 0 | 0 | 0 | 0 | 0 | 0 | 0 | 0 | 0 | 1 |
| *Amelanchier* sp. | 0 | 0 | 0 | 0 | 0 | 0 | 0 | 0 | 0 | 0 | 0 | 1 | 0 |
| *Antennaria plantaginifolia* | 1 | 0 | 0 | 0 | 0 | 0 | 0 | 0 | 0 | 0 | 0 | 1 | 0 |
| *Aquilegia canadiensis* | 0 | 0 | 1 | 0 | 0 | 0 | 0 | 0 | 0 | 0 | 1 | 1 | 1 |
| *Arabis* sp. | 0 | 0 | 0 | 0 | 0 | 0 | 0 | 1 | 0 | 0 | 0 | 0 | 0 |
| *Brassica* sp*.* | 0 | 0 | 1 | 0 | 0 | 0 | 0 | 0 | 0 | 0 | 0 | 0 | 0 |
| *Chionanthus virginicus* | 0 | 0 | 0 | 1 | 0 | 0 | 0 | 0 | 0 | 0 | 0 | 0 | 0 |
| *Erodium cicutarium* | 1 | 0 | 0 | 0 | 0 | 0 | 0 | 0 | 0 | 0 | 0 | 0 | 0 |
| *Geranium robertianum* | 0 | 0 | 0 | 0 | 0 | 0 | 0 | 0 | 0 | 0 | 0 | 0 | 1 |
| *Hesperis matronalis* | 0 | 0 | 0 | 0 | 0 | 0 | 0 | 0 | 0 | 1 | 0 | 0 | 1 |
| *Lamia* sp | 1 | 0 | 0 | 0 | 0 | 0 | 0 | 0 | 0 | 0 | 0 | 0 | 0 |
| *Lonicera sempervirens* | 0 | 0 | 0 | 1 | 0 | 0 | 0 | 0 | 0 | 0 | 0 | 0 | 0 |
| *Lonicera tatarica* | 0 | 0 | 0 | 0 | 0 | 0 | 0 | 0 | 0 | 1 | 0 | 0 | 1 |
| *Oxalis stricta* | 0 | 0 | 0 | 0 | 0 | 0 | 0 | 0 | 1 | 0 | 0 | 0 | 0 |
| *Phlox subulata* | 0 | 0 | 0 | 0 | 0 | 0 | 0 | 1 | 0 | 0 | 0 | 0 | 0 |
| *Potentilla norvegica* | 0 | 0 | 0 | 0 | 0 | 0 | 0 | 0 | 0 | 0 | 1 | 0 | 1 |
| *Ranunculus* sp. | 0 | 1 | 0 | 1 | 0 | 0 | 0 | 0 | 0 | 0 | 0 | 0 | 1 |
| *Saponnaria officinalis* | 0 | 0 | 0 | 0 | 0 | 0 | 0 | 1 | 0 | 0 | 0 | 0 | 0 |
| *Saxifraga paniculata* | 0 | 0 | 0 | 0 | 0 | 0 | 0 | 0 | 0 | 0 | 0 | 1 | 0 |
| *Senecio jacobaea* | 0 | 0 | 0 | 0 | 0 | 0 | 0 | 0 | 1 | 0 | 0 | 0 | 0 |
| *Silene* sp. | 0 | 0 | 0 | 0 | 1 | 1 | 0 | 0 | 0 | 0 | 0 | 0 | 0 |
| *Silene virginica* | 0 | 0 | 0 | 1 | 0 | 0 | 0 | 0 | 0 | 0 | 0 | 0 | 0 |
| *Vaccinium corymbosum* | 0 | 0 | 0 | 0 | 1 | 0 | 0 | 0 | 0 | 0 | 0 | 0 | 0 |
| *Vaccinium angustifolium* | 0 | 0 | 1 | 1 | 0 | 0 | 0 | 0 | 0 | 0 | 0 | 1 | 0 |
| *Veronica* sp | 1 | 0 | 0 | 0 | 0 | 0 | 0 | 0 | 0 | 0 | 0 | 0 | 0 |
| *Viola bicolor* | 1 | 0 | 0 | 0 | 0 | 0 | 0 | 0 | 0 | 0 | 0 | 0 | 0 |

**Table S8** Comparisons based on the Akaike information criterion (AIC) between mixed-effects models testing for an association of pollinator service with range position: latitude (Model 1); latitude and its square term (Model 2); latitude and elevation (Model 3); latitude, its square term and elevation (Model 4); and intercept only (Model 0). The number of replicates (*N*) is the number of original observations, camera and day for pollinator data, or population (13), patch (166), flower measured (520) or day recording (39) for mechanistic variables.

|  |  |  |  | AIC | | | | | | |
| --- | --- | --- | --- | --- | --- | --- | --- | --- | --- | --- |
|  |  | Model 0 |  | Model 1 |  | Model 2 |  | Model 3 |  | Model 4 |
| A. Pollinator service | *N* |  |  |  |  |  |  |  |  |  |
| *Visitation rate* | 382 | 1921 |  | 1919 |  | 1924 |  | 1930 |  | 1934 |
| *Pollination ratio* | 382 | 100 |  | 102 |  | 112 |  | 120 |  | 128 |
| *Pollinator richness* | 382 | 1468 |  | 1472 |  | 1477 |  | 1485 |  | 1490 |
| *Shannon index* | 382 | 518 |  | 524 |  | 533 |  | 541 |  | 548 |
|  |  |  |  |  |  |  |  |  |  |  |
| B. Mechanistic variables |  |  |  |  |  |  |  |  |  |  |
| *Population size* (log_10_) | 13 | 36.6 |  | 32.1 |  | 34.0 |  | 33.9 |  | 35.9 |
| *Local flower density* (log_10_) | 166 | 157.2 |  | 163.8 |  | 171.5 |  | 179.8 |  | 186.9 |
| *Flower size* | 520 | 3203.5 |  | 3204.2 |  | 3206.2 |  | 3213.5 |  | 3214.2 |
| *Plant sp. richness* | 13 | 58.1 |  | 59.3 |  | 49.8 |  | 57.3 |  | 51.8 |
| *Mean T°* | 39 | 209.5 |  | 210.7 |  | 213.4 |  | 218.8 |  | 221.6 |

**Table S9** Table of means and standard errors of variables depicting pollinator service for the 13 *Arabidopsis lyrata* populations studied

| Pop |  | Flowers recorded |  | Visitation rate | |  | Pollination ratio | |  | Richness | |  | Shannon index | |
| --- | --- | --- | --- | --- | --- | --- | --- | --- | --- | --- | --- | --- | --- | --- |
|  |  |  |  | [visits per flower & day] | |  | [visited to total flowers] | |  | [sp. per patch & day ] | |  |  |  |
|  |  |  |  | Mean | SE |  | Mean | SE |  | Mean | SE |  | Mean | SE |
| NC1 |  | 668 |  | 2.23 | 0.34 |  | 0.63 | 0.05 |  | 3.18 | 0.34 |  | 0.79 | 0.09 |
| NC2 |  | 484 |  | 0.55 | 0.15 |  | 0.35 | 0.05 |  | 0.94 | 0.15 |  | 0.14 | 0.05 |
| NC3 |  | 714 |  | 1.54 | 0.20 |  | 0.65 | 0.05 |  | 2.75 | 0.33 |  | 0.72 | 0.10 |
| NC4 |  | 401 |  | 1.65 | 0.24 |  | 0.51 | 0.05 |  | 1.88 | 0.25 |  | 0.42 | 0.08 |
| VA1 |  | 1446 |  | 2.22 | 0.21 |  | 0.75 | 0.04 |  | 4.47 | 0.42 |  | 1.01 | 0.10 |
| VA2 |  | 468 |  | 0.90 | 0.15 |  | 0.49 | 0.07 |  | 2.58 | 0.25 |  | 0.62 | 0.09 |
| MD1 |  | 82 |  | 1.31 | 0.28 |  | 0.46 | 0.08 |  | 1.00 | 0.23 |  | 0.20 | 0.07 |
| WV1 |  | 122 |  | 5.59 | 1.65 |  | 0.79 | 0.06 |  | 2.20 | 0.27 |  | 0.53 | 0.10 |
| MD4 |  | 325 |  | 3.46 | 0.47 |  | 0.88 | 0.03 |  | 2.30 | 0.24 |  | 0.56 | 0.10 |
| PA2 |  | 1338 |  | 2.30 | 0.23 |  | 0.80 | 0.03 |  | 3.50 | 0.21 |  | 0.88 | 0.06 |
| NY3 |  | 484 |  | 2.49 | 0.24 |  | 0.84 | 0.04 |  | 2.81 | 0.33 |  | 0.69 | 0.10 |
| NY4 |  | 435 |  | 5.73 | 0.50 |  | 0.87 | 0.03 |  | 4.90 | 0.43 |  | 1.15 | 0.10 |
| NY6 |  | 348 |  | 4.21 | 0.83 |  | 0.76 | 0.05 |  | 1.80 | 0.19 |  | 0.40 | 0.06 |

**Table S10** Variance inflation factor (VIF) values of the models testing for associations between pollinator services and the various mechanistic variables: *A. lyrata* population size, local flower density, flower size, flowering plant species richness and mean temperature

|  |  | Population size (log_10_) |  | Local flower density (log_10_) |  | Local flower density^2^ (log_10_) |  | Flower size |  | Plant sp. richness |  | Mean T° |
| --- | --- | --- | --- | --- | --- | --- | --- | --- | --- | --- | --- | --- |
|  |  | VIF |  | VIF |  | VIF |  | VIF |  | VIF |  | VIF |
| Pollinator service | | |  |  |  |  |  |  |  |  |  |  |
| *Visitation rate* | | 1.055 |  | 1.029 |  | 1.002 |  | 1.074 |  | 1.075 |  | 1.017 |
| *Pollination ratio* | | 1.083 |  | 1.585 |  | 1.522 |  | 1.049 |  | 1.075 |  | 1.020 |
| *Richness* |  | 1.073 |  | 1.452 |  | 1.403 |  | 1.047 |  | 1.070 |  | 1.016 |
| *Shannon index* | | 1.073 |  | 1.423 |  | 1.375 |  | 1.048 |  | 1.071 |  | 1.018 |
|  |  |  |  |  |  |  |  |  |  |  |  |  |


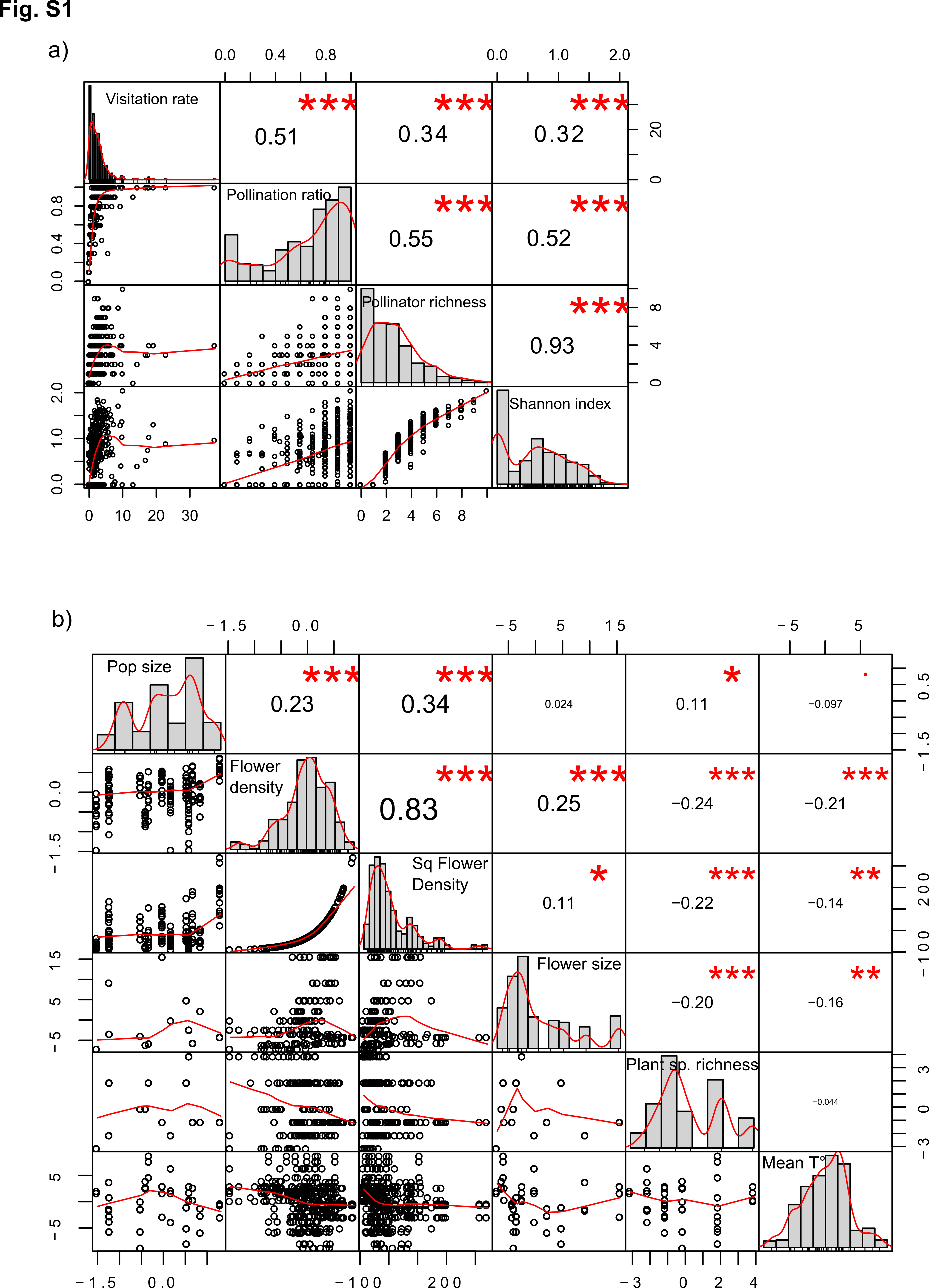


**Fig. S1** Matrices of pairwise correlations between estimates of pollinator service (A) and mechanistic variables (B). Cells along the diagonals contain histograms, those of the lower triangles scatter plots with predictions from a locally-fit polynomial model, and those of the upper triangles Pearson correlation coefficients (with significance indicated by stars).

**S1.** R codes of mixed-effects models testing for an association between pollinator service and range position (Code Question 1), and for an association between pollinator service and potential mechanistic variables (Code Question 2)

Code Question 1: *Does pollinator service decline from the centre toward range edges?*

e.g., *Model=lmer (visitation rate ~ latitude,*

*+ (1 | population)*

*+ (1 | population:camera:year),*

*control=lmerControl(optimizer="bobyqa", optCtrl = list(maxfun = 1e5)),*

*data=Data, na.action=na.omit)*

Code Question 2. *What are the mechanisms for reduced pollinator service?*

*Model=lmer (visitation rate ~ population size + local flower density + local flower density*^2^

*flower size + plant sp. richness + mean temperature*

*+ (1 | Population)*

*+ (0 + local flower density | population)*

*+ (0 + local flower density*^2^ *| population)*

*+ (0 + mean temperature| population)*

*+ (1 | population: camera: year),*

*control=lmerControl(optimizer="bobyqa", optCtrl = list(maxfun = 1e5)),*

*data=Data, na.action=na.omit)*
